# HIV-1 Vpr mediates the depletion of the cellular repressor CTIP2 to counteract viral gene silencing

**DOI:** 10.1038/s41598-019-48689-x

**Published:** 2019-09-11

**Authors:** F. Forouzanfar, S. Ali, C. Wallet, M. De Rovere, C. Ducloy, H. El Mekdad, M. El Maassarani, A. Aït-Ammar, J. Van Assche, E. Boutant, F. Daouad, F. Margottin-Goguet, C. Moog, C. Van Lint, C. Schwartz, O. Rohr

**Affiliations:** 1 Université de Strasbourg, EA7292, FMTS, IUT Louis Pasteur, Schiltigheim, France; 2 Institute of Microbiology, University of Agriculture, Faisalabad, Pakistan; 3 INSERM U1109, Fédération de Médecine Translationnelle (FMTS), Université de Strasbourg, Strasbourg, France; 4 Université de Strasbourg, UMR 7021 CNRS, Illkirch, France; 5 Inserm, U1016, Institut Cochin, 22 rue Méchain, 75014 Paris, France; 6 CNRS, UMR8104, Paris, France; 7 Université Paris Descartes, Sorbonne Paris Cité, Paris, France; 8 Université Libre de Bruxelles (ULB), Service of Molecular Virology, Department of Molecular Biology (DBM), Gosselies, Belgium

**Keywords:** Infection, Transcriptional regulatory elements

## Abstract

Mammals have evolved many antiviral factors impacting different steps of the viral life cycle. Associated with chromatin-modifying enzymes, the cellular cofactor CTIP2 contributes to HIV-1 gene silencing in latently infected reservoirs that constitute the major block toward an HIV cure. We report, for the first time, that the virus has developed a strategy to overcome this major transcriptional block. Productive HIV-1 infection results in a Vpr-mediated depletion of CTIP2 in microglial cells and CD4+ T cells, two of the major viral reservoirs. Associated to the Cul4A-DDB1-DCAF1 ubiquitin ligase complex, Vpr promotes CTIP2 degradation via the proteasome pathway in the nuclei of target cells and notably at the latent HIV-1 promoter. Importantly, Vpr targets CTIP2 associated with heterochromatin-promoting enzymes dedicated to HIV-1 gene silencing. Thereby, Vpr reactivates HIV-1 expression in a microglial model of HIV-1 latency. Altogether our results suggest that HIV-1 Vpr mediates the depletion of the cellular repressor CTIP2 to counteract viral gene silencing.

## Introduction

Jawed vertebrates including mammals have developed innate and acquired immune responses to fight infections. Mammals have evolved many antiviral factors such as SAMHD1 or APOBEC3G that limit virus expression^1–3^. We have reported that the cellular cofactor CTIP2 (Bcl11b) favors the establishment and the persistence of HIV-1 post-integration latency in microglial cells, the main target of the virus in the brain^4–6^. CTIP2 works as a scaffold protein to recruit at least two different complexes in microglial cells. As part of a chromatin remodelling complex CTIP2 is associated with the lysine demethylase LSD1, the histone deacetylases HDAC1 and HDAC2, and the histone methyltransferase SUV39H1^4,7–9^. In CD4+ T cells, CTIP2 associates with the NuRD chromatin remodelling complex^10^. In addition to this epigenetic silencing, CTIP2 contributes to control the elongation process of gene transcription. Indeed, we found CTIP2 associated with an inactive P-TEFb complex including HEXIM1, HMGA1 and the 7SK snRNA^11,12^. As part of this new complex, CTIP2 represses P-TEFb functions and thereby contributes to limit the reactivation of latently integrated HIV-1 proviruses. Altogether, CTIP2 favors the establishment and the persistence of HIV-1 gene latency in microglial cells, the major reservoirs of virus in the central nervous system.

Lentiviruses such as SIV and HIV have developed several mechanisms to evade innate and adaptive immune responses. “Accessory” proteins (Nef, Vif, Vpu, Vpx) are encoded by these viruses to allow immune evasion. For example, Vpx is involved in the degradation of SAMHD1 via the proteasome pathway (reviewed in^13^). Proteasome-mediated degradation is a privileged pathway subverted by HIV-1 to counteract cellular defenses. Associated with a Cul4A-based ubiquitin ligase complex through DCAF1 (VprBP), the HIV-1 accessory protein Vpr has long been hypothesized to target an as-yet undiscovered host restriction factor for degradation in order to induce G2 arrest^14,15^. Vpr has been proposed to promote the degradation of cellular factors including UNG2^16^, Dicer^17^, IRF3^18^, ZIP^19^, the HTLF DNA translocase^20^ and the post-replication DNA repair factor Exo1^21^. By activation of the SLX4 complex, Vpr prevents an appropriate innate immune response^22^. Recently, Vpr has been shown to repress HIV-1 expression by targeting APOBEC3G^23^ and an as-yet unidentified macrophage restriction factor^24^. Finally, two recent papers described that the human silencing hub complex (HUSH) is also targeted to degradation by Vpr and HIV-2/SIV Vpx^25,26^. Interestingly, the HUSH complex is presented by the authors as a potent restriction factor regulating the transcription step of the viral life cycle.

Here we show that HIV-1 developed a strategy to counteract CTIP2-mediated repression. We report that the viral accessory protein Vpr promotes the degradation of CTIP2 through physical and functional interactions with the Cul4A-DDB1-DCAF1 complex. Vpr targets CTIP2 associated with the heterochromatin-modifying complex. Interestingly, Vpr expression also correlated with a reduced binding of CTIP2 at the HIV-1 promoter and a reactivation of the latent virus in the CHME5-HIV microglial model of latency.

## Results

### Hiv-1 Expression promotes a vpr-dependent degradation of ctip2

To investigate a possible modulation of CTIP2 expression in response to HIV-1 infection, we measured its protein and its RNA levels in time course experiments in Jurkat T-cells and microglial cells.

**Figure 1:**
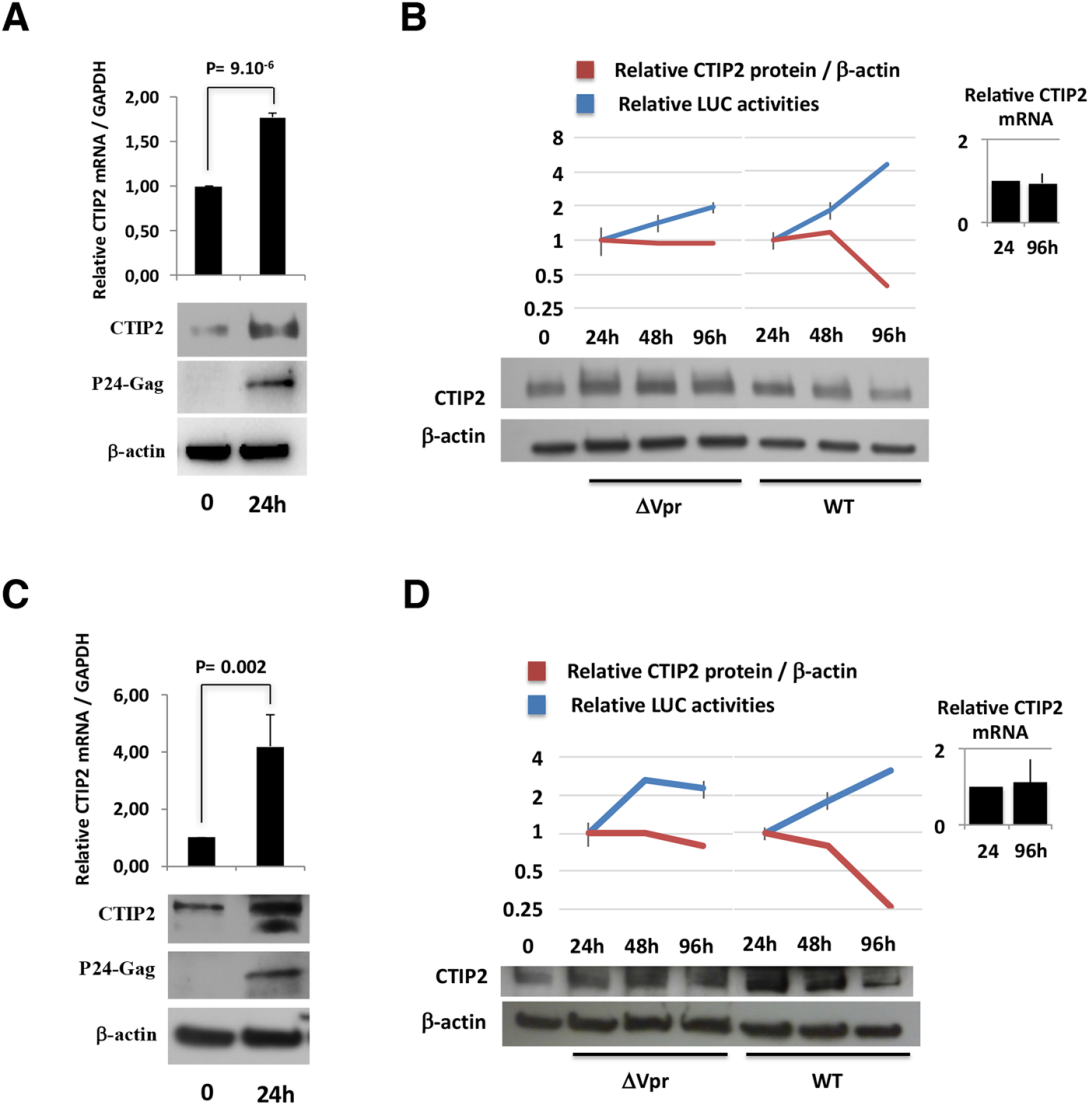
CTIP2-induced expression in infected cells is counteracted by HIV-1 in a Vpr dependent manner. (**A**,**C**) Jurkat T cells (**A**) and Microglial cells (**C**) were infected with 25 × 10^3^ copies/ml of a VSV-G pseudotyped NL4.3ΔEnv-LUC provirus for 24 h. The presence of CTIP2 in infected cells was assessed by western blot and the CTIP2 mRNA quantified by RT-Q-PCR. (**B**,**D**) Jurkat T cells (**B**) and Microglial cells (**D**) were infected with 150 × 10^3^ copies/ml of a VSV-G pseudotyped NL4.3ΔEnv-LUC (WT) and a NL4.3ΔEnvΔVpr -LUC (ΔVpr) provirus for 24, 48 and 96 h. The presence of CTIP2 in infected cells was assessed by western blot and the viral expression was quantified by luciferase assay. The quantifications are presented relative to the quantities obtained after 24 h of infection. The results are representative of at least three independent experiments.

**Figure 2:**
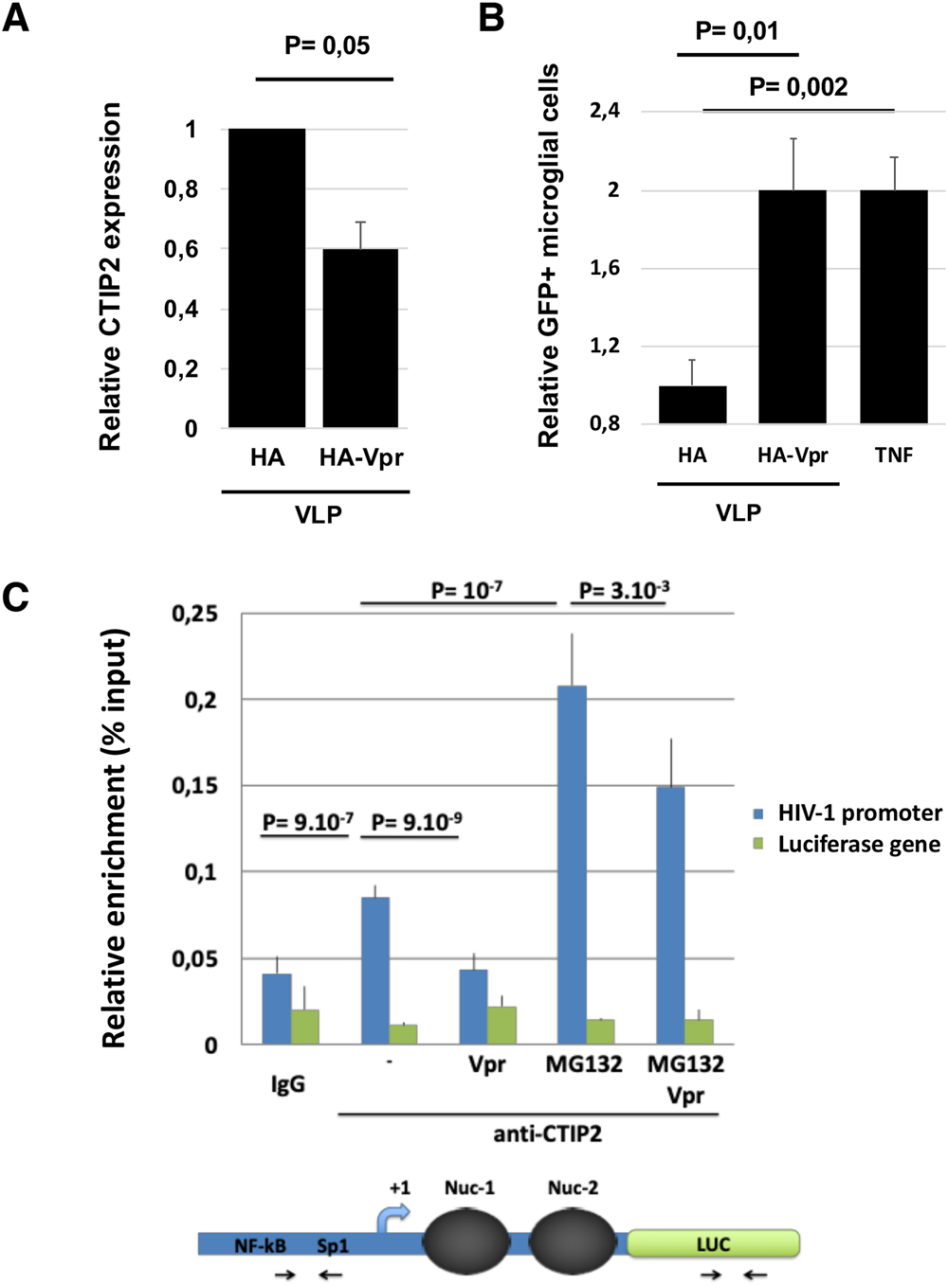
Vpr promotes depletion of CTIP2 in primary CD4+ T cells and at the HIV-1 promoter to induce viral reactivation in microglial cells. (**A**) Relative CTIP2 expression in CD4 T cells was quantified by flow cytometry. Expression levels in cells incubated with Virus Like-Particles VLP-HA-Vpr were compared to the control VLP-HA particles taken as 1. (**B**) CHME5-HIV latently infected microglial cell line was infected with VPLs or treated by TNFα. The number of GFP+ cells was quantified by flow cytometry and presented relative to GFP+ cells obtained after VLP-HA infections. (**C**) Chromatin Immuno-precipitation experiments targeting endogenous CTIP2 at the HIV-1 promoter were performed with chromatin from microglial cells expressing the chromatinized LTR-LUC episomal vector and Vpr as indicated. The cells were subjected or not to MG132 treatment. Enrichments are presented as percentages of the inputs. As a control, the presence of CTIP2 has been concomitantly quantified at the luciferase gene region. Results are representative of at least three independent experiments.

**Figure 3:**
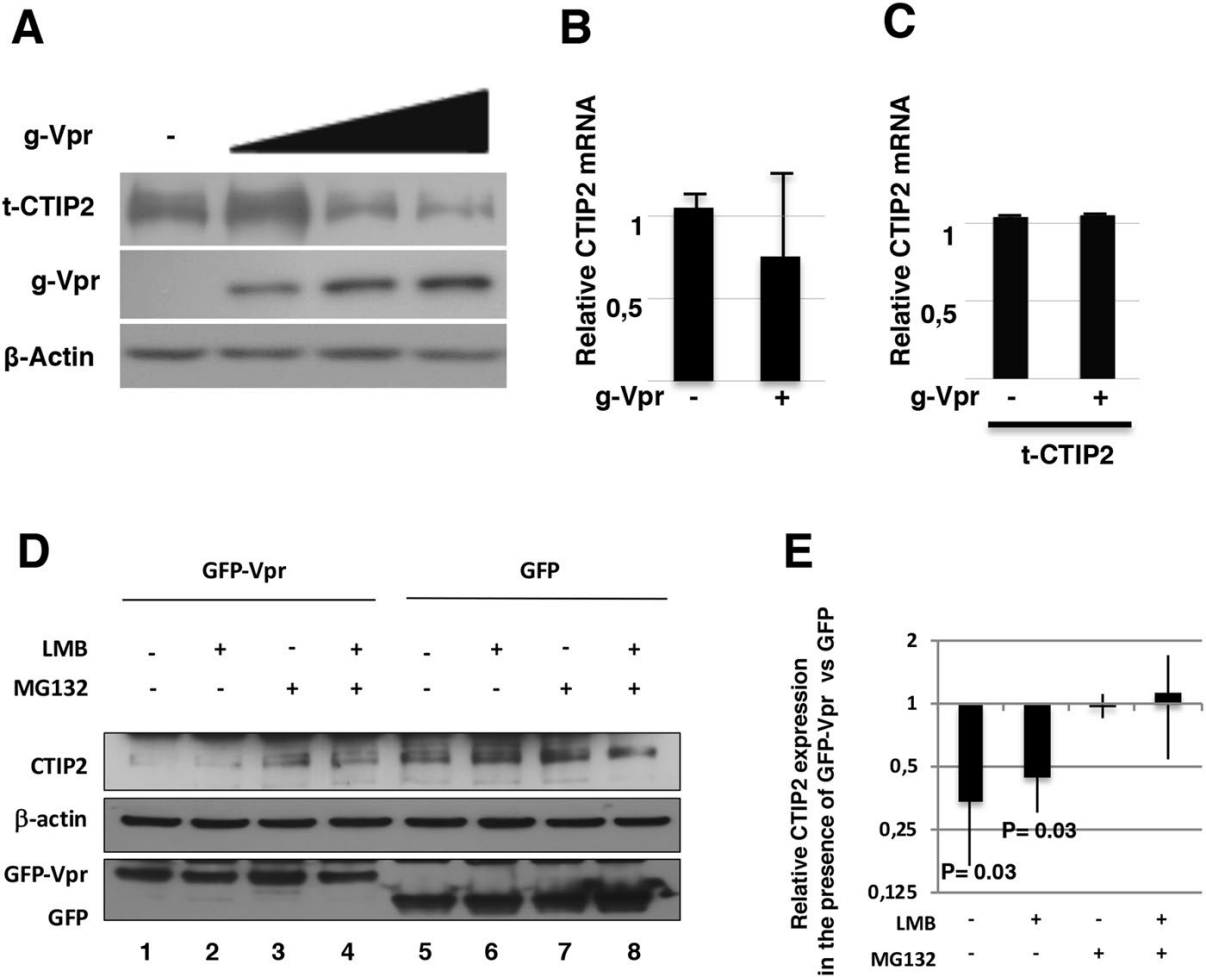
Vpr targets nuclear CTIP2 to degradation via the proteasome pathway. (**A**) Nuclear extracts from HEK cells expressing Tap-CTIP2 (t-CTIP2) and increasing amount of GFP-Vpr (g-Vpr) were analyzed by western blot for the presence of the proteins indicated. (**B**,**C**) CTIP2 mRNA were quantified by RT-Q-PCR in the presence or not of Vpr in the absence (**B**) and in the presence (**C**) of CTIP2 overexpression. The results are presented relative to the pCDNA3 control vector taken as one. (**D**) Endogenous CTIP2 expression was analyzed by western blot in extracts from cells expressing GFP-Vpr or the control GFP and subjected to the indicated treatments. Cells were treated with 10 ng/ml leptomycin B for 4 h and then with MG132 (5 μg/μl) for 6 h before lysis and nuclear protein extraction. Protein expression was assessed by western blot. (**E**) Expression of CTIP2 in the presence of GFP-Vpr was quantified relative to β-actin and presented relative to the quantities obtained in the presence of the GFP control protein^57^. Results are representative of at least three independent experiments.

Following an initial induction of both CTIP2 mRNA and protein expression after the first 24 h post HIV-1-infection (Fig. 1, panels A and C), CTIP2 protein expression levels decreased in Jurkat T cells (Fig. 1B WT panel) and in microglial cells (Fig. 1D, WT panel) at later time points. Interestingly, while levels of CTIP2 mRNA were not affected along the time course (from 24 h to 96 h of infection), deletion of the Vpr gene (Vpr-deleted virus - ΔVpr) abrogated CTIP2 depletion upon viral expression (Fig. 1B,D, ΔVpr Panels). To confirm that the depletion of CTIP2 mediated by Vpr occurs at a post-transcriptional step, we next overexpressed a FLAG tagged CTIP2 in the presence of normalized quantities of WT and ΔVpr proviruses. The WT-, but not the ΔVpr provirus, promoted a depletion of CTIP2 expressed under the control of a CMV promoter (Figure S1). These results confirmed that CTIP2 expression was impacted post-transcriptionally. Vpr molecules are known to be incorporated into the virions^27^. To determine if incoming Vpr proteins are sufficient to induce a depletion of CTIP2 in primary cells, we next generated virus-like particles (VLPs) to deliver Vpr in purified CD4+ T cells. As shown in Fig. 2A, incoming Vpr induced a significant depletion of CTIP2 in primary CD4+ T cells, suggesting that Vpr produced in cells upon HIV-1 replication and Vpr delivered by the virions upon infection reduce CTIP2 expression. Since CTIP2 has been described to limit the reactivation of latently integrated viruses in microglial cells^9^, we next investigated the impact of incoming Vpr on the CHME5-HIV microglial model of HIV-1 latency. Interestingly, Vpr delivery increased by two-fold the number of HIV-1 expressing cells (Fig. 2B). This magnitude of reactivation was similar to that obtained with TNFα treatment (Fig. 2B column HA-Vpr vs TNFα). As we previously reported that CTIP2 associates with the latent HIV-1 promoter in microglial cells, we next performed chromatin IP experiments targeting CTIP2 in the presence or not of Vpr (Fig. 2C). As expected, CTIP2 was found associated with the viral promoter but not with the luciferase gene of the construct in microglial cells (Fig. 2C: IgG vs (-) mock CTIP2 IP column). However, Vpr expression significantly reduced the binding of CTIP2 (Fig. 2C: Vpr vs (-) mock), in line with the previously observed depletion of the protein in nuclear extracts. Vpr has been shown to interact directly with the 26 S proteasome and to degrade target cellular factors via the proteasome pathway^28^. We indeed found CTIP2 associated with the 19S subunit of the proteasome but not Vpr (Figure S5). To further determine the mechanism underlying CTIP2 depletion, we next treated the cells with the proteasome inhibitor MG132. As shown, MG132 treatment favored CTIP2 binding to the viral promoter (MG132 vs (-) mock) and strongly impaired the impact of Vpr (MG132+ Vpr vs MG132). Altogether, these results suggest that Vpr produced in cells upon HIV-1 replication and Vpr particles incorporated in virions both contribute to the depletion of CTIP2 in cell lines and primary cells. This depletion correlates with reduced binding of CTIP2 at the viral promoter and reactivation of the latent HIV-1. In addition, the impact of MG132 on Vpr-mediated depletion of CTIP2 and the interaction of CTIP2 with the 19S subunit of the proteasome suggest an involvement of the proteasome pathway and a degradation of the protein rather than translational regulation. To further investigate Vpr-induced depletion of CTIP2 biochemically, we next expressed increasing amounts of Vpr in CTIP2 expressing cells. As expected, Vpr expression promoted a dose-dependent depletion of CTIP2 (Fig. 3A). Of note, levels of CTIP2 mRNA from control and from CTIP2-overexpressing cells were not significantly affected by Vpr (Fig. 3B,C, respectively) confirming the post-transcriptional impact of Vpr on CTIP2 expression. To further investigate the involvement of the proteasome pathway, microglial cells were treated with the proteasome inhibitor MG132. In accordance with the ChIP results presented in Fig. 2C, MG132 increased the level of endogenous CTIP2 expression in nuclear extracts (Fig. 3D, row 7 vs 5), suggesting that the physiological turnover of CTIP2 needs a functional proteasome pathway. Again, Vpr reduced CTIP2 expression (Fig. 3D, column 5 vs 1), and MG132 impaired Vpr-mediated depletion of CTIP2 (Fig. 3D, column 1 vs 3). These observations strongly advocate for a degradation of CTIP2 by the proteasome pathway. To determine if this degradation occurs in the nucleus, cells were treated with leptomycin B (LMB), an inhibitor of nuclear export. As shown, LMB treatment did not significantly affect the level of CTIP2 degradation by Vpr (Fig. 3D, column 2 vs 1). We next took advantage of the replicates of these experiments to quantify the impact of Vpr on CTIP2 expression levels in the presence of LMB and MG132. As shown in Fig. 3E, Vpr promoted a more than two-fold depletion of CTIP2 in microglial cells nuclei (first column). However, MG132 abolished the Vpr-mediated depletion of CTIP2 in the presence or absence of LMB (two last columns). Altogether, our results suggest that Vpr degrades CTIP2 via the proteasome pathway in the nuclei of HIV-1 target cells.

### Ctip2 Interacts with the cull4/ddb1-dcaf1 ubiquitin ligase complex

**Figure 4:**
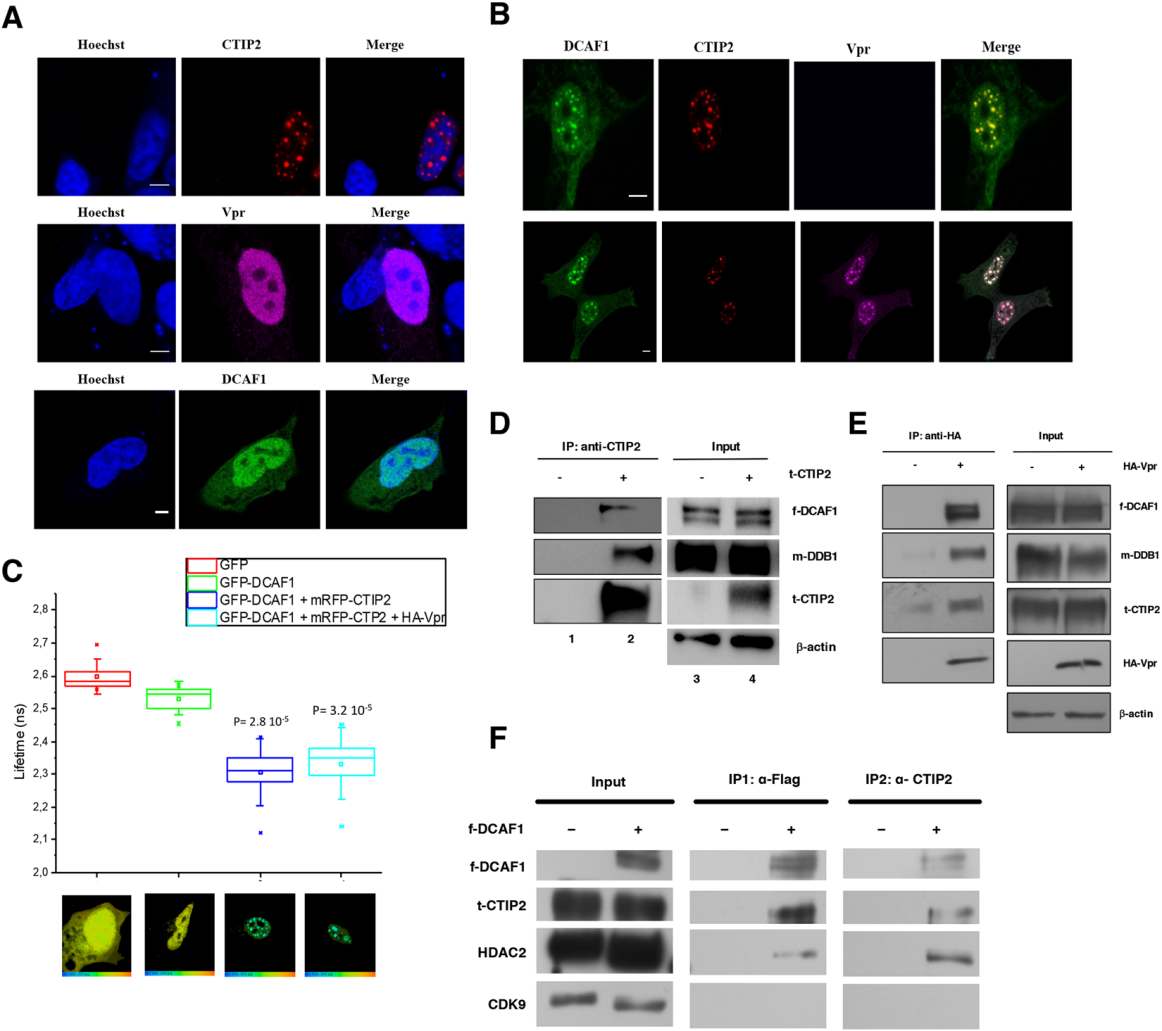
CTIP2 interacts with the Cull4-DDB1-DCAF1 ubiquitin ligase complex. (**A**,**B**) Microglial cells expressing RFP-CTIP2, HA-Vpr and GFP-DCAF1 alone (panel A) or in combination (panel B) were subjected to confocal microscopy. Scale bars are set to 5 µm. (**C**) Lifetimes from GFP proteins FRET experiments are presented for each condition. The results are representative of 5 independent experiments and from 6 to 10 quantifications for each condition per experiment. (**D**,**E**) Nuclear extracts from cells expressing the proteins indicated were subjected to immunoprecipitation experiments targeting Tap-CTIP2 (t-CTIP2) (**D**) and HA-Vpr (**E**). The inputs and the immunoprecipitated complexes were analyzed by western blot. (**F**) Nuclear extracts from cells expressing the proteins indicated were subjected to a first immunoprecipitation targeting FLAG-DCAF1 (f-DCAF) with anti-FLAG antibodies. The DCAF1 associated complexes were eluted by incubation with FLAG peptides and subjected to a second immuno-precipitation targeting CTIP2. The presence of the indicated proteins was analyzed at each step by western blot.

Vpr has been reported to target cellular proteins for degradation through recruitment of the Cul4A-DDB1-DCAF1 complex. Since inhibiting the proteasome pathway increased CTIP2 expression levels in the absence of Vpr, we first investigated its possible interaction with the ubiquitin ligase complex. Confocal microscopy observations were performed on microglial cells expressing RFP-CTIP2, GFP-DCAF1, and HA-Vpr alone (Fig. 4A) or in combination (Fig. 4B). As previously described, CTIP2 is localized in dense- and structured-nuclear domains^29^. The expression of HA-Vpr and GFP-DCAF1 was mainly observed in the nuclear compartment. However, the combined expression of RFP-CTIP2 with GFP-DCAF1 promoted a relocation of DCAF1 proteins to CTIP2-induced structures (Fig. 4B, upper panel). The yellow staining of the CTIP2-induced structures results from this colocation. Interestingly, Vpr expression further turned the staining of CTIP2 structures white, suggesting a triple colocation of Vpr, DCAF1 and CTIP2 (Fig. 4B, lower panel). We have previously reported that CTIP2 associates with Vpr in CTIP2-induced nuclear structures^7^. To determine whether CTIP2 interacts directly with DCAF1, we next performed fluorescence resonance energy transfer (FRET) experiments. As shown in Fig. 4C, the presence of RFP-CTIP2 reduced significantly the lifetime of GFP-DCAF1 in the nuclear structures, indicating a direct interaction between these two proteins. Surprisingly, the presence of Vpr did not significantly impact on this effect. To confirm the physical interaction between DCAF1 and CTIP2 biochemically, we performed co-immuno-precipitation experiments targeting first CTIP2. DCAF1 and DDB1 were found in association with CTIP2 (Fig. 4D). These associations are in accordance with the increased CTIP2 expression observed following MG132 treatment. In addition, we found Vpr associated with CTIP2 and the DDB1-DCAF1 complex in nuclear extracts from MG132-treated cells (Fig. 4E). These results suggest that Vpr takes advantage of the cellular degradation machinery to deplete CTIP2 from HIV-1 infected cells and favor HIV-1 expression.

We have reported that CTIP2 associates with two different and exclusive complexes: A complex dedicated to epigenetic silencing of gene expression (chromatin modifying enzymes)^9^ and an inactive form of the P-TEFb elongation complex including the 7SK snRNA^11^. To characterize CTIP2-associated DCAF1 containing complexes, we performed sequential immunoprecipitation (IP) experiments (Fig. 4F). In the first IP that targeted DCAF1-associated complexes, we found CTIP2 and HDAC2 (chromatin-modifying enzyme) but not CDK9, the kinase subunit of the P-TEFb complex. This result suggests that DCAF1 may interact with CTIP2 and its chromatin-modifying complex. To confirm that CTIP2, HDAC2 and DCAF1 are part of the same complex, proteins were eluted from the first IP and submitted to a second IP targeting CTIP2. DCAF1 and HDAC2 were found associated with CTIP2 in the eluted DCAF1-containing complex, suggesting that CTIP2, DCAF1 and HDAC2 are members of the same complex (Fig. 4F). Altogether, these results strongly suggest that DCAF1 interacts with and degrades CTIP2 bound to the chromatin modifying enzyme complex (HDAC1/2, SUV39H1) but not with CTIP2 bound to the inactive P-TEFb complex. This conclusion is in accordance with the degradation of HDAC1 by Vpr upon HIV-1 infection (Figure S3 and^30^).

### Association with dcaf1 is crucial for vpr-mediated degradation of ctip2

**Figure 5:**
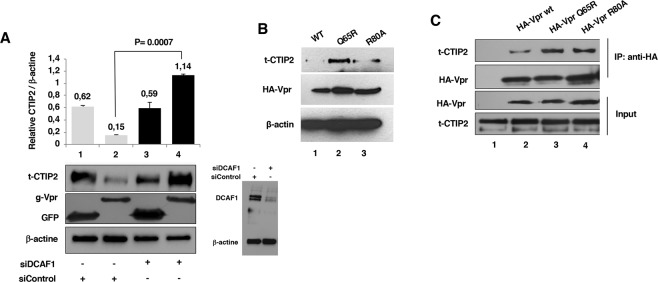
DCAF1 binding is necessary for Vpr-mediated degradation of CTIP2. (**A**) Nuclear extracts from control or DCAF1 knock-down cells were analyzed by western blot for the presence of the indicated proteins in the presence of GFP-Vpr and the control GFP. CTIP2 expression was quantified relative to the loading control using ImageJ software. DCAF1 knock-down efficiency in our experiments is presented. (**B**) Nuclear extracts from cells expressing the indicated proteins were analyzed by western blot. β-actin is presented as a loading control. (**C**) Nuclear extracts from cells expressing large amount of Tap-CTIP2 (t-CTIP2) and the indicated HA-Vpr proteins were subjected to immunoprecipitation experiments targeting the HA tag. The input and the Vpr-associated complexes were analyzed for the presence of CTIP2 by western blot.

To further characterize mechanistically the degradation of CTIP2 by Vpr, we explored whether DCAF1 is needed for the Vpr-mediated degradation process by using a Si RNA-based knock-down approach. As expected, depletion of DCAF1 abrogated the degradation of CTIP2 (Fig. 5A column 4 vs 2). We next quantified CTIP2 expression levels in the presence of WT or mutated Vpr proteins (Fig. 5B). The mutations Q65R (which abrogates DCAF1 binding and G2 arrest) and R80A (supports efficient DCAF1 binding but abolishes G2 arrest) were tested^14,31,32^. As expected, CTIP2 was barely detectable in the presence of WT and R80A Vpr proteins (Fig. 5B columns 1 and 3). However, the Q65R mutation, abolished Vpr-mediated degradation of CTIP2 (Fig. 5B column 2). These results demonstrated that Vpr-mediated G2 arrest does not impact CTIP2 expression and confirmed the requirement for DCAF1 for the depletion of CTIP2 by Vpr. Interestingly, upon a strong overexpression of CTIP2, allowing its detection in the presence of Vpr, we were able to find WT, R80A and Q65R Vpr proteins associated with CTIP2 (Fig. 5C). Thereby, our results suggest that recruitment of DCAF1 is crucial to ensure the Vpr-mediated degradation of CTIP2 but dispensable for the physical interactions between the proteins.

## Discussion

CTIP2/Bcl11B is involved both in the establishment and the persistence of HIV-1 latency in CD4+ T cells and microglial cells^8,9,11,33^. By silencing integrated HIV-1 proviruses and disfavoring its reactivation, this cellular cofactor constitutes a major block to viral expression. To prevent abortive infections, HIV-1 has evolved sophisticated mechanisms to evade innate and adaptive immune responses. Accessory proteins such as Nef, Vif, Vpu, Vpx and Vpr are dedicated to some aspects of this immune evasion. Indeed, several restriction factors are targeted to degradation by these accessory proteins. Vif targets APOBEC3G^23^, Vpu targets tetherin by sequestration from the site of budding and proteasomal degradation^34,35^ and Vpx targets SAMHD1^2^. Among the so-called accessory proteins which have to be rather considered as key virulence factors, Vpr remained enigmatic. Vpr is a small protein (around 14 kDa) which is involved in several biological functions including nuclear import of the pre-integration complex^36^; envelope protein expression and virion production^24^; HIV-1 transcription^37^; post-replication DNA repair^21^, HIV-1 gene splicing^38^; induction of apoptosis^39^ and cell cycle arrest^22^ (reviewed in^40^). Vpr is produced upon HIV-1 replication and a significant amount (275 molecules) is incorporated in the new virion^27^. We report here that Vpr expressed in infected cells (Fig. 1) and incoming-Vpr molecules (Fig. 2) favor the degradation of CTIP2 as previously described for the translocase HTLF^20^. Interestingly, incoming Vpr is necessary to overcome the HIV-1 transcriptional block observed in dendritic cells^41^. The influence of CTIP2 in dendritic cells has not yet been studied. However, our results suggest that Vpr contributes to unlock CTIP2-mediated blocks of HIV-1 gene expression. Vpr has been described associated with the E3 ubiquitin ligase complex to promote a proteasome-mediated degradation of cellular targets (for review^15^). Here we report that CTIP2 interacts with this ubiquitin ligase complex via DCAF1/VprBP. Of note, DCAF1 was reported to interact and to protect Vpr from proteasomal degradation^42^. We found Vpr, CTIP2 and DCAF1 co-localized in dense subnuclear structures, and FRET studies demonstrated a direct association between CTIP2 and DCAF1. These observations suggested to us that Vpr may subvert the proteasome pathway to promote CTIP2 degradation. Interestingly, we showed that CTIP2 protein expression is higher in cells treated with MG132 suggesting a physiological regulation of CTIP2 through the proteasome pathway. Such a CTIP2 protein degradation has been described in a physiological condition such as TCR activation^43,44^. Therefore, Vpr promotes a constitutive DCAF1-dependent CTIP2 turnover. Such a mechanism of action for Vpr has already been described for UNG2^16^ and discussed elsewhere^15^. In agreement with this, we show that Vpr enhances the ubiquitination of CTIP2 (Figure S2). An increase of the basal ubiquitination has already been described for HDAC1 and HDAC3, proteins associated with the formation of heterochromatin, the compact-inactive form of the promoters^30,45^. We further report that Vpr promotes CTIP2 degradation in the nucleus, at the HIV-1 promoter. This observation strongly argues for an indirect impact of Vpr on HIV-1 gene transcription. As a consequence of CTIP2 degradation, Vpr contributes to overcome the transcriptional silencing induced by CTIP2-associated complexes, and to favors Tat function (Figure S4). Interestingly, we found CTIP2, HDAC2 and DCAF1 in the same complex. Since P-TEFb is excluded from this complex, our results suggest that Vpr promotes the degradation of CTIP2 associated with HDAC1/2 and SUV39H1. The concomitant degradation of CTIP2 and HDAC1 (Figure S3) and the recent demonstration that Vpr targets HDAC1 to a proteasome-mediated degradation further support these conclusions^30^. Moreover, results of a recent screen for factors targeted by the virulence factors Vpr and HIV-2/SIV Vpx strengthen our results^25,26^. This screen identified the human silencing hub (HUSH) complex as a new target of Vpr^46,47^. By the degradation of CTIP2 and its associated heterochromatin-promoting enzymes, Vpr unlocks an epigenetic block and activates HIV-1 gene transcription. As commented in^48^, our results suggest a link between the intrinsic immunity and the epigenetic control of HIV-1 gene transcription, which might lead to new ways to combat HIV-1.

There are now several pieces of evidence that Vpr is a key virulence factor. It has been shown to disturb several cellular pathways including cell cycle arrest (reviewed in^40^) and DNA repair (reviewed in^49^). The involvement of Vpr in the latter pathway has been associated with the viral escape from innate immune sensing^22^. We and others reported that Vpr is involved in the epigenetic regulation of HIV-1 gene transcription. Vpr promotes the degradation of members of chromatin remodeling complexes such as CTIP2, HDAC1^30^, ZIP and sZIP, the adaptors of the NuRD complex^19^. Interestingly CTIP2 recruits NuRD to silence HIV-1 gene transcription in CD4+ T cells^10^. Recently, we have shown that PKC-mediated phosphorylation on Ser2 of CTIP2 abrogates the recruitment of NuRD upon activation via the TCR^50^. Post-translational modifications drive the association of CTIP2 with different complexes and thereby determine CTIP2 functions. Whether or not Vpr is able to discriminate between the diverse forms of CTIP2 will require further investigation.

The recognition of Vpr functions in the viral escape from the immune responses in general and in preventing HIV-1 latency, as described in the present article, may have profound therapeutic implications^51^. We have recently reviewed the possibilities of targeting post-replication DNA repair complexes to prevent HIV-1 latency^52^. Taking advantage of Vpr-induced depletions of key cellular factors may be an interesting option, as recently described for the post replication DNA repair protein Exo1^21^.

## Materials and Methods

### Plasmids

Most of the constructs used in our assays have been described previously: pcDNA3, pNTAP, pFLAG-CTIP2 (f-CTIP2), pRFP-CTIP2, pNTAP-CTIP2 (t-CTIP2) were used^8,9,11^. pGFP-Vpr WT (g-Vpr), pNL- 4.3 ∆Env-luc wt, pNL-4.3 ∆Env-luc ∆Vpr^19^ and pMyc-DDB1 (m-DDB1), pFLAG-DCAF1 (f-DCAF1), pHA- Vpr wt, pHA-Vpr Q65R were kindly provided by Dr. F. Margottin-Goguet.

### Cell culture

The human microglial (provided by M. Tardieu, Paris, France)^53^, HEK293T and CHME5-HIV cell lines were maintained in Dulbecco’s modified Eagle medium (DMEM) and Jurkat cell line in RPMI 1640. The culture mediums were completed with 10% (vol/vol) heat-inactivated fetal bovine serum (FBS) and 100 U/mL penicillin/streptomycin. Where indicated, the cells were treated with MG132 (5 μg/μl) for 6 h, 10.0 ng/ml leptomycin B (LMB) for 5 h before harvesting the cells.

### Antibodies and reagents

CTIP2 (ab18465) antibodies were purchased from Abcam, BCL11b (A300–384A) from Bethyl Laboratories, CDK9 (sc-8338), Myc epitope (9 E10), mouse and rat HRP-conjugated from Santa Cruz Biotechnology, anti-FLAG M2 affinity gel and β-actin (A5441) from Sigma-Aldrich, and anti-HA.11 Tag Antibody (AFC-101P) was from Eurogentec. Rabbit HRP-conjugated antibodies were purchased from Thermo Fisher Scientific, anti-GFP (632592) from Clontech, and Protein G Magnetic beads from Millipore. MG132 and leptomycin B molecules were provided by Sigma-Aldrich, and the protease inhibitor mixture by Roche. VprBP (DCAF1) and non-targeting siRNAs were from Dharmacon. SYBR Select Master Mix used for Q-PCR was from Thermo Fisher. CTIP2-FITC (25B6) ab123449 and IgG control were purchased from Abcam. Anti-HA.11-AF647 was used for the confocal microscopy observations.

### HIV-1 infections and viral production in cell lines

The VSV-G pseudotyped ΔEnv-LUC HIV-1 viruses were produced by transfecting the pNL4.3 ΔEnv-LUC HIV-1 (WT) or pNL4.3 ΔEnvΔVpr-LUC HIV-1 (ΔVpr) constructs together with the VSV-G expression plasmid in 293 T cells. 48 h after transfection, the culture supernatants were collected and viral production was measured by clinical HIV-1 viral load test (quantification of the genomic RNAs).

### CTIP2 expression in primary CD4+ T cells infected with VLP

Primary CD4+ T cells were isolated and purified from human peripheral blood mononuclear cells (PBMC) of healthy blood donors by positive selection using CD4 MicroBeads selection kits and autoMACS Pro Separator (Miltenyi Biotec) as previously described^54,55^. CD4^+^ T lymphocytes were activated with phytohemaglutinin A (PHA) (2 µg/ml) (Sigma-Aldrich) for 3 days in RPMI 1640 plus Gibco GlutaMAX medium (Thermo Fisher) supplemented with 10% fetal calf serum (FCS). Cells were stored frozen. They were thawed the night before use. CD4 T cells were incubated with Viral-like particles VLP-HA or VLP-HA-Vpr (obtained from FMG) at 0.1 µg for 0.1 million cells for 1 days. For the quantification of intracellular CTIP2 expression, cells were incubated for 10 min at 4 °C with CD3 cell surface molecules before fixation with the Cytofix solution (BD Biosciences) and permeabilized with a solution of 0.1% Triton X-100. CTIP2 or IgG control isotype were incubated for 30 min at 4 °C. After extensive washing, the percentage of CD3-PEC T cells positive for CTIP2 expression was defined by flow cytometry (MACSQuant, Miltenyi) and analyzed with Kaluza software.

### Co-immunoprecipitation assays and western blotting

Cells cultured in 150-mm diameter dishes were transfected using a calcium phosphate co-precipitation method with the plasmids indicated. Lysates were prepared two days post-transfection as previously described^11^. Immunoprecipitations were performed using the standard technique, as in^11^. Finally, the immunoprecipitated complexes were processed for SDS-PAGE and immunoblot analysis. Proteins were visualized by chemiluminescence using the SuperSignal Chemiluminescence Detection System (Thermo Fisher).

### Chromatin immunoprecipitation (ChIP)

Cells expressing the chromatinized HIV-1 episomal LTR-luciferase construct were subjected to ChIP experiments with IgG control or anti-CTIP2 IgG antibodies, as in^8,9^. The presence of CTIP2 at the promoter- or the luciferase gene- regions was quantified by qPCR. Results are presented as percentages of inputs. Values are representative of three independent experiments.

### Confocal microscopy

Microglial cells were transfected in 24-well glass-bottomed plates (Greiner Bio-One, Ref. 662892) with a DNA mixture containing eGFP-DCAF1, mRFP-CTIP2 or HA-Vpr. At 24 h post-transfection, cells were extensively washed, fixed with 4% PFA/PBS solution and kept in 1x PBS at 4 °C until observation with a Leica SPE confocal microscope equipped with a Leica 63x1.4NA oil immersion objective (HXC PL APO 63x/1.40 OIL CS).

### Immunofluorescence staining

Microglial cells expressing HA-tagged Vpr were immunodetected as described before^56^. Anti-HA (BioLegend, 901501) was diluted 1/1000 and Alexa Fluor goat anti-mouse (A-21237, Thermo Fisher) 1/250. Cells were analyzed by confocal microscopy.

### Fluorescence lifetime imaging microscopy (FLIM)

Time-correlated single-photon counting FLIM was performed on a home-made laser scanning set-up based on an Olympus IX70 inverted microscope with an Olympus 60× 1.2NA water immersion objective, as described^56^. Two-photon excitation at 900 nm was provided by an InSight DeepSee laser (Spectra Physics). For FLIM, the laser power was adjusted to give count rates with peaks up to as few as 10^6^ photons.s^−1^, so that the pile-up effect can be neglected. Photons were collected using a short pass filter with a cut-off wavelength of 680 nm (F75-680, AHF, Germany), and a band-pass filter of 520 ± 17 nm (F37-520, AHF, Germany). The fluorescence was directed to a fiber-coupled APD (SPCM-AQR-14-FC, Perkin Elmer), which was connected to a time-correlated single photon counting module (SPC83, Becker and Hickl, Germany). Typically, the samples were scanned continuously for about 60 s to achieve appropriate photon statistics to analyze the fluorescence decays. The FRET phenomenon causes a decrease in the fluorescent lifetime (τ) of the donor, which can be measured by the FLIM technique at each pixel or group of pixels. Experiments were performed with eGFP as a FRET donor and mRFP as acceptor. The FRET efficiency (E) was calculated according to: E = 1 − τ_DA_/τ_D_ = 1/1 + (R/R_0_)^6^ where τ_DA_ is the lifetime of the donor in the presence of the acceptor and τ_D_ is the lifetime of the donor in the absence of the acceptor. A Förster R_0_ distance (where the transfer efficiency is 50%) of 56 Ä was calculated between eGFP used as a donor and mRFP used as acceptor.

### Luciferase assays

Microglial cells cultured in 48-well plates were transfected with the indicated vectors and the CMV-Renilla luciferase control vector using the calcium phosphate co-precipitation method. Two days later, cells were collected and firefly luciferase activity was determined using the Dual-Glo Luciferase Assay System (Promega, Madison, WI, USA) and normalized to the Renilla luciferase activity. Values correspond to an average of at least three independent experiments performed in triplicates.

### mRNA quantification

The total RNA was extracted using an RNeasy Plus Mini Kit (Qiagen, Germantown, MD, USA) according to the manufacturer’s instructions. Using SuperScript III Reverse Transcriptase (Thermo Fisher) and oligo (dT)12–18 Primers (Thermo Fisher), RNA was reverse transcribed into cDNA. The cDNA was diluted 10-fold with DNase-free water. Quantitative PCR was performed on the diluted cDNA using SYBR Select Master Mix. Primers used in qPCR include: CTIP2-F 5′-ATTGGAACCTGCCACTTG-3′, CTIP2-R 5′-TTTGCCTGTGTTCCACGA-3′ GAPDH-F5′-GAGAAGGCTGGGGCTCATTT-3′, GAPDH-R 5′-GCAGTGATGGCATGGACTGT-3′. Data were normalized to GAPDH and calculated using the 2^−(ΔΔCT)^ method.

### Statistical analysis

Data were statistically analyzed by using *t* tests with Excel (Microsoft) or StatView (SAS Institute Inc.). A value of *p* < 0.05 was considered statistically significant. Results were expressed as S.D. and represent data from a minimum of three independent experiments.

## Supplementary information

Supplementary Informations

## References

[1] Descours B (2012). SAMHD1 restricts HIV-1 reverse transcription in quiescent CD4(+) T-cells. Retrovirology.

[2] Laguette N (2011). SAMHD1 is the dendritic- and myeloid-cell-specific HIV-1 restriction factor counteracted by Vpx. Nature.

[3] Lahouassa H (2012). SAMHD1 restricts the replication of human immunodeficiency virus type 1 by depleting the intracellular pool of deoxynucleoside triphosphates. Nat. Immunol..

[4] Le Douce V, Cherrier T, Riclet R, Rohr O, Schwartz C (2014). The Many Lives of CTIP2: From AIDS to Cancer and Cardiac Hypertrophy. J. Cell. Physiol..

[5] Le Douce V (2016). HIC1 controls cellular- and HIV-1- gene transcription via interactions with CTIP2 and HMGA1. Sci. Rep..

[6] Marban C (2016). Targeting the Brain Reservoirs: Toward an HIV Cure. Front. Immunol..

[7] Cherrier T (2009). p21(WAF1) gene promoter is epigenetically silenced by CTIP2 and SUV39H1. Oncogene.

[8] Le Douce V (2012). LSD1 cooperates with CTIP2 to promote HIV-1 transcriptional silencing. Nucleic Acids Res..

[9] Marban C (2007). Recruitment of chromatin-modifying enzymes by CTIP2 promotes HIV-1 transcriptional silencing. Embo J.

[10] Cismasiu VB (2005). BCL11B functionally associates with the NuRD complex in T lymphocytes to repress targeted promoter. Oncogene.

[11] Cherrier T (2013). CTIP2 is a negative regulator of P-TEFb. Proc Natl Acad Sci USA.

[12] Eilebrecht S (2014). HMGA1 recruits CTIP2-repressed P-TEFb to the HIV-1 and cellular target promoters. Nucleic Acids Res..

[13] Mashiba M, Collins KL (2013). Molecular mechanisms of HIV immune evasion of the innate immune response in myeloid cells. Viruses.

[14] Le Rouzic E (2007). HIV1 Vpr arrests the cell cycle by recruiting DCAF1/VprBP, a receptor of the Cul4-DDB1 ubiquitin ligase. Cell Cycle.

[15] Romani B, Cohen EA (2012). Lentivirus Vpr and Vpx accessory proteins usurp the cullin4-DDB1 (DCAF1) E3 ubiquitin ligase. Curr. Opin. Virol..

[16] Wen X, Casey Klockow L, Nekorchuk M, Sharifi HJ, de Noronha CMC (2012). The HIV1 protein Vpr acts to enhance constitutive DCAF1-dependent UNG2 turnover. PLoS One.

[17] Casey Klockow L (2013). The HIV-1 protein Vpr targets the endoribonuclease Dicer for proteasomal degradation to boost macrophage infection. Virology.

[18] Okumura A (2008). HIV-1 accessory proteins VPR and Vif modulate antiviral response by targeting IRF-3 for degradation. Virology.

[19] Maudet C (2013). HIV-1 Vpr induces the degradation of ZIP and sZIP, adaptors of the NuRD chromatin remodeling complex, by hijacking DCAF1/VprBP. PLoS One.

[20] Lahouassa H (2016). HIV-1 Vpr degrades the HLTF DNA translocase in T cells and macrophages. Proc. Natl. Acad. Sci. USA.

[21] Yan, J. *et al*. HIV-1 Vpr Reprograms CLR4 ^DCAF1^ E3 Ubiquitin Ligase to Antagonize Exonuclease 1-Mediated Restriction of HIV-1 Infection. *MBio* **9** (2018). 10.1128/mBio.01732-18 PMC6199497 30352932

[22] Laguette N (2014). Premature activation of the SLX4 complex by Vpr promotes G2/M arrest and escape from innate immune sensing. Cell.

[23] Zhou Dawei, Wang Yan, Tokunaga Kenzo, Huang Fang, Sun Binlian, Yang Rongge (2015). The HIV-1 accessory protein Vpr induces the degradation of the anti-HIV-1 agent APOBEC3G through a VprBP-mediated proteasomal pathway. Virus Research.

[24] Mashiba M, Collins DR, Terry VH, Collins KL (2014). Vpr overcomes macrophage-specific restriction of HIV-1 Env expression and virion production. Cell Host Microbe.

[25] Yurkovetskiy Leonid, Guney Mehmet Hakan, Kim Kyusik, Goh Shih Lin, McCauley Sean, Dauphin Ann, Diehl William E., Luban Jeremy (2018). Primate immunodeficiency virus proteins Vpx and Vpr counteract transcriptional repression of proviruses by the HUSH complex. Nature Microbiology.

[26] Chougui G (2018). HIV-2/SIV viral protein X counteracts HUSH repressor complex. Nat. Microbiol..

[27] Müller B, Tessmer U, Schubert U, Kräusslich HG (2000). Human immunodeficiency virus type 1 Vpr protein is incorporated into the virion in significantly smaller amounts than gag and is phosphorylated in infected cells. J. Virol..

[28] Li G (2010). HIV-1 replication through hHR23A-mediated interaction of Vpr with 26S proteasome. PLoS One.

[29] Rohr O (2003). Recruitment of Tat to heterochromatin protein HP1 via interaction with CTIP2 inhibits human immunodeficiency virus type 1 replication in microglial cells. J Virol.

[30] Romani B, Baygloo NS, Hamidi-Fard M, Aghasadeghi MR, Allahbakhshi E (2016). HIV-1 Vpr Protein Induces Proteasomal Degradation of Chromatin-associated Class I HDACs to Overcome Latent Infection of Macrophages. J. Biol. Chem..

[31] DeHart JL (2007). HIV-1 Vpr activates the G2 checkpoint through manipulation of the ubiquitin proteasome system. Virol. J..

[32] Lv L (2018). Vpr Targets TET2 for Degradation by CRL4 VprBP E3 Ligase to Sustain IL-6 Expression and Enhance HIV-1 Replication. Mol. Cell.

[33] Cismasiu VB (2008). BCL11B is a general transcriptional repressor of the HIV-1 long terminal repeat in T lymphocytes through recruitment of the NuRD complex. Virology.

[34] Mitchell RS (2009). Vpu antagonizes BST-2-mediated restriction of HIV-1 release via beta-TrCP and endo-lysosomal trafficking. PLoS Pathog..

[35] Douglas JL (2009). Vpu directs the degradation of the human immunodeficiency virus restriction factor BST-2/Tetherin via a {beta}TrCP-dependent mechanism. J. Virol..

[36] Popov S (1998). Viral protein R regulates nuclear import of the HIV-1 pre-integration complex. EMBO J..

[37] Kino T (2002). Human immunodeficiency virus type 1 (HIV-1) accessory protein Vpr induces transcription of the HIV-1 and glucocorticoid-responsive promoters by binding directly to p300/CBP coactivators. J. Virol..

[38] Kuramitsu M (2005). A novel role for Vpr of human immunodeficiency virus type 1 as a regulator of the splicing of cellular pre-mRNA. Microbes Infect..

[39] Nishizawa M, Kamata M, Mojin T, Nakai Y, Aida Y (2000). Induction of apoptosis by the Vpr protein of human immunodeficiency virus type 1 occurs independently of G(2) arrest of the cell cycle. Virology.

[40] Guenzel CA, Hérate C, Benichou S (2014). HIV-1 Vpr-a still &quot;enigmatic multitasker&quot. Front. Microbiol..

[41] Miller CM (2017). Virion-Associated Vpr Alleviates a Postintegration Block to HIV-1 Infection of Dendritic Cells. J. Virol..

[42] Le Rouzic E (2008). Assembly with the Cul4A-DDB1^DCAF1^ Ubiquitin Ligase Protects HIV-1 Vpr from Proteasomal Degradation. J. Biol. Chem..

[43] Zhang L (2012). Coordinated Regulation of Transcription Factor Bcl11b Activity in Thymocytes by the Mitogen-activated Protein Kinase (MAPK) Pathways and Protein Sumoylation. J. Biol. Chem..

[44] Selman WH, Esfandiari E, Filtz TM (2019). Alteration of Bcl11b upon stimulation of both the MAP kinase- and Gsk3-dependent signaling pathways in double-negative thymocytes. Biochem. Cell Biol..

[45] Romani B (2016). HIV-1 Vpr reactivates latent HIV-1 provirus by inducing depletion of class I HDACs on chromatin. Sci. Rep..

[46] Robbez-Masson L (2018). The HUSH complex cooperates with TRIM28 to repress young retrotransposons and new genes. Genome Res..

[47] Tchasovnikarova IA (2015). Epigenetic silencing by the HUSH complex mediates position-effect variegation in human cells. Science (80-.)..

[48] Van Lint C (2018). Stop HUSHing on SIV/HIV. Nat. Microbiol..

[49] Brégnard C, Benkirane M, Laguette N (2014). DNA damage repair machinery and HIV escape from innate immune sensing. Front. Microbiol..

[50] Dubuissez M (2016). Protein Kinase C-Mediated Phosphorylation of BCL11B at Serine 2 Negatively Regulates Its Interaction with NuRD Complexes during CD4+ T-Cell Activation. Mol. Cell. Biol..

[51] Schwartz Christian, Bouchat Sophie, Marban Céline, Gautier Virginie, Van Lint Carine, Rohr Olivier, Le Douce Valentin (2017). On the way to find a cure: Purging latent HIV-1 reservoirs. Biochemical Pharmacology.

[52] Schwartz C, Rohr O, Wallet C (2018). Targeting the DNA-PK complex: its rationale use in cancer and HIV-1 infection. Biochem. Pharmacol..

[53] Janabi N, Peudenier S, Héron B, Ng KH, Tardieu M (1995). Establishment of human microglial cell lines after transfection of primary cultures of embryonic microglial cells with the SV40 large T antigen. Neurosci. Lett..

[54] Su B (2012). Neutralizing antibodies inhibit HIV-1 transfer from primary dendritic cells to autologous CD4 T lymphocytes. Blood.

[55] Holl V (2010). Stimulation of HIV-1 Replication in Immature Dendritic Cells in Contact with Primary CD4 T or B Lymphocytes. J. Virol..

[56] Fritz JV (2008). Direct Vpr-Vpr interaction in cells monitored by two photon fluorescence correlation spectroscopy and fluorescence lifetime imaging. Retrovirology.

[57] McDonald D, Carrero G, Andrin C, de Vries G, Hendzel MJ (2006). Nucleoplasmic beta-actin exists in a dynamic equilibrium between low-mobility polymeric species and rapidly diffusing populations. J. Cell Biol..

