# Supplementary material for: HIV-1 Vpr mediates the depletion of the cellular repressor CTIP2 to counteract viral gene silencing
Source: Sci Rep. 2019 Sep 11;9:13154. doi: 10.1038/s41598-019-48689-x (PMC6739472; doi:10.1038/s41598-019-48689-x)
Supplement: Supplementary file 1 — Supplementary Informations [file 41598_2019_48689_MOESM1_ESM.pdf]

SUPPLEMENTARY INFORMATIONS

HIV-1 Vpr mediates the depletion of the cellular repressor CTIP2 to counteract viral gene silencing

Forouzanfar F.[1], Ali S.[1,2], Wallet C.[1], De Rovere M[1]., Ducloy C.[3], El Mekdad H.[1], El Maassarani M.[1], Aït-Ammar A.[1,8], Van Assche J[1]., Boutant E. [4], Daouad F. [1], Margottin-Goguet F. [5,6,7], Moog C. [3], Van Lint C. [8*], Schwartz C. [1*] and Rohr O[1*].

[1] Université de Strasbourg, EA7292, FMTS, IUT Louis Pasteur, Schiltigheim, France.
[2] Institute of Microbiology, University of Agriculture, Faisalabad, Pakistan.
[3] INSERM U1109, Fédération de médecine translationnelle (FMTS), Université de Strasbourg, Strasbourg, France ;
[4] Université de Strasbourg, UMR 7213 CNRS, Illkirch, France ;
[5] Inserm, U1016, Institut Cochin, 22 rue Méchain, 75014 Paris, France
[6] CNRS, UMR8104, Paris, France
[7] Université Paris Descartes, Sorbonne Paris Cité, Paris, France
[8] Université Libre de Bruxelles (ULB), Service of Molecular Virology, Department of Molecular Biology (DBM), Gosselies, Belgium.

[*]Correspondence to olivier.rohr@unistra.fr; christian.schwartz@unistra.fr; cvlint@ulb-ac.be

Lead contact: Olivier ROHR
olivier.rohr@unistra.fr

Figure S1

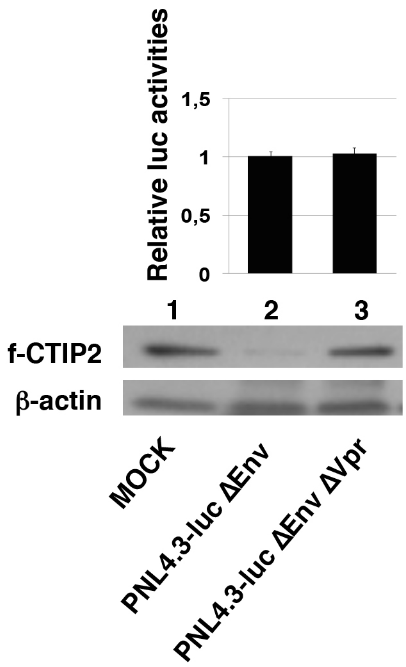

Figure S1: The WT- but not the ΔVpr provirus, promotes a strong depletion of CTIP2
Cells expressing FLAG-CTIP2 and normalized quantities of NL4.3ΔENV-LUC and NL4.3ΔEnvΔVpr –LUC viruses were analyzed for CTIP2 expression by western blot and the expression of the two viruses by luciferase assay. β-actin is presented as a loading control. The results are representative of at least three independent experiments.

Figure S2

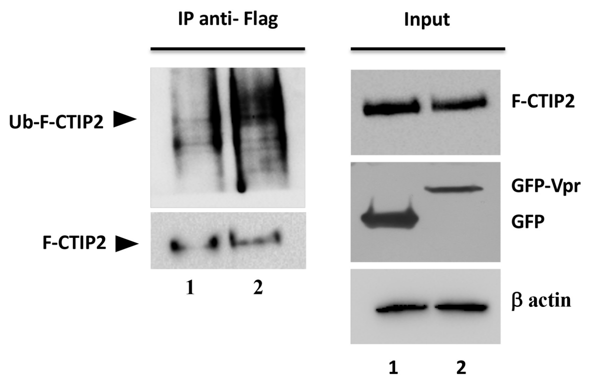

Figure S2: CTIP2 is ubiquitylated in the presence of Vpr.
Nuclear extracts from MG132-treated cells expressing FLAG-CTIP2, HA-Ub and GFP (column 1) or GFP-Vpr (column 2) were subjected to an anti-FLAG IP and a western blot targeting ubiquitin. Inputs show expression levels of the proteins indicated.

Figure S3

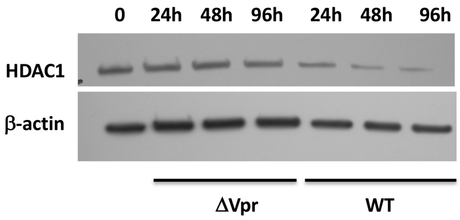

Figure S3: HDAC1 and CTIP2 are concomitantly depleted by HIV-1 in a Vpr-dependent manner

Jurkat T cells were infected with 150 000 copies/ml of a VSV-G pseudotyped NL4.3ΔEnv-LUC (WT) and a NL4.3ΔEnvΔVpr -LUC (ΔVpr) provirus for 24, 48 and 96 h. The presence of HDAC1 in infected cells have been assessed by western blot. CTIP2 expression levels at the same time points are presented in Figure 1.

Figure S4

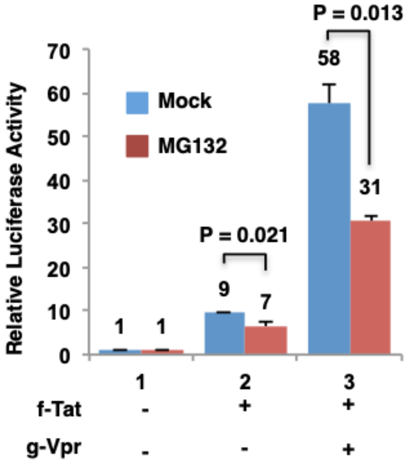

Figure S4: HIV-1 Vpr favors Tat-mediated transactivation
Cells expressing the LTR-LUC construct and the viral proteins indicated were treated or not with proteasome inhibitor MG132 and analyzed for the activity of the viral promoter by luciferase assay. The results are representative of at least three independent experiments performed in triplicate.

Figure S5

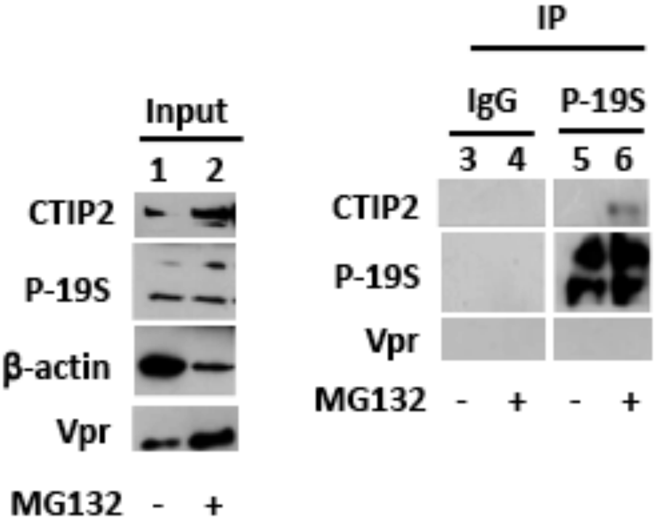

Figure S5: CTIP2 associates with the 19S subunit (P-19S) of the 26S Proteasome complex.

Nuclear extracts from MG132 treated and mock treated cells expressing FLAG-CTIP2 and HA-Vpr were subjected to immunoprecipitation with anti-19S subunit of the proteasome complex (column 5 and 6) or control IgG antibodies (column 3 and 4). The presence of the indicated proteins was assessed by western blot. Cropped images are from the same gel and the same western-blot membrane.
